# The Effect of Material Type and Location of an Orthodontic Retainer in Resisting Axial or Buccal Forces

**DOI:** 10.3390/ma14092319

**Published:** 2021-04-29

**Authors:** Jaana Ohtonen, Lippo Lassila, Eija Säilynoja, Pekka K. Vallittu

**Affiliations:** 1 BioCity Turku Biomaterials and Medical Device Research Program, Department of Biomaterials Science, Institute of Dentistry, University of Turku, 20520 Turku, Finland; liplas@utu.fi (L.L.); pekval@utu.fi (P.K.V.); 2 City of Kotka Municipal Health Centre, 48600 Kotka, Finland; 3 Turku Clinical Biomaterials Centre—TCBC, Institute of Dentistry, University of Turku, 20520 Turku, Finland; 4 GC Group, Stick Tech Ltd., 20520 Turku, Finland; eija.sailynoja@gc.dental; 5 City of Turku Welfare Division, 20520 Turku, Finland

**Keywords:** orthodontic FRC retainer, HS glass, composite, stainless steel, mechanical test

## Abstract

The purpose of this study was to investigate the effect of retainer material and retainer position on a tooth to resist movement of the tooth in a simulation model. Bidirectional continuous glass fiber-reinforced composite (FRC) retainers and control retainers of steel wires were tested. The FRC retainers had a polymer matrix of bisphenol-A-glycidyldimethacrylate (bis-GMA) and poly(methylmethacrylate) (PMMA), and it was cured with a photoinitiator system. The retainers were adhered to a lower jaw Frasaco model in two different positions. Resistance against the movement of one tooth was measured from two directions. The average load values within the FRC retainer groups were higher than within the metal retainer groups. The load values for the groups loaded from the axial direction were higher than those loaded from the buccal direction. FRC retainers, which were located 1–2 mm from the incisal edge, showed higher load values than those located 4–5 mm from the incisal edge. There was a significant difference in load values between FRC retainers and metal retainers (*p* < 0.01). The wire position and the direction of force also had significant effects (*p* < 0.01). There were no significant differences between metal retainer groups. The results of this study suggest that metal retainers are more flexible, allowing for tooth movements of larger magnitude than with FRC retainers.

## 1. Introduction

Orthodontic retention has an important role in orthodontic treatment for maintaining an achieved outcome. After orthodontic treatment, periodontal and gingival ligaments need time to reorganize. Growth may also change the treatment results; if a patient’s teeth are in an unstable position, retention must be permanent or treatment will relapse. Proper retention is needed to prevent unwanted, possible relapse from occurring [1,2,3,4]. Retention devices, such as retainers, can be fixed or removable [5]. Fixed retainers in the lower jaw are typically bonded to all six front teeth or only to the canines [5,6]. Typically, fixed retainers are made from monofilament stainless steel wire (0.030 or 0.032 inches, corresponding to 0.752 or 0.0813 mm), multistranded round stainless steel [7,8], or flattened eight-braided stainless steel wires [9,10].

Along with metal wires, fiber-reinforced composite (FRC) retainers are another option for fixed retention. The matrix of a typical dental FRC consists of a combination of monomers such as bisphenol-A-glycidyl dimethacrylatere (Bis-GMA) and triethyleneglycol dimethacrylate (TEGDMA), and additional polymers such as poly-(methyl methacrylate) (PMMA). The polymer matrix is reinforced by fibers [11]. Fibers in orthodontic retainers are often polyethylene, E-glass, or S-glass fibers [11,12,13,14], and they are protected by the polymer matrix. The proper adhesion of fibers to the polymer matrix is important because it allows stress transfer from the polymer matrix to the fibers and, thus, affects the physical properties of the composite [15]. Metal and FRC retainers are both bonded to the enamel using regular adhesive techniques. Multistranded stainless steel retainers are still the most popular material for lingual retainers [1], since they are known to allow the bonded teeth to move slightly. The FRC retainers are fully covered by a flowable resin composite, which thickens the retainer and lowers the flexibility of the retainer. This may have an influence on the retainer’s longevity, especially with regard to the bonding durability of the retainer [12,16]. Placement of multistranded steel retainers is a relatively simple process for orthodontists. However, the placement of FRC retainers may be more challenging, which may also be a reason for the higher number of failures with FRC retainers compared to that of multistranded steel retainers [16].

The lower incisors provide vertical space to place the retainer, but in the case of upper incisors in normal and, especially, in deep bite situations, the space in occlusion is limited, which may cause loosening of the retainers [17]. Multistranded stainless steel wires are thin (0.0215 or 0.175 inches, corresponding to 0.55 or 0.45 mm) and flattered eight-braided wires are even thinner (0.016 × 0.026 or 0.028 inches × 0.008 inches, corresponding to 0.41 × 0.66 or 0.71 mm × 0.20 mm), whereas an FRC retainer with an overlaying flowable resin composite may become thicker, being, e.g., 0.75 mm (everStick Ortho^®^ 0.75 mm, Angelus Interlig^®^ 0.2 mm, Ribbond THM-Ortho^®^ 0.18 mm × 1.0 mm). A layer of flowable resin composite, which is used to cover the FRC core, is required to prevent wear of the FRC layer [18].

The purpose of this study was to investigate the resistance of retainers of different types of wear to allow tooth movement of a 0.1 mm magnitude, which was used as an indicator for clinical flexibility of the bonded retainer. The load required to move a tooth in the buccolingual and axial directions was measured. In addition, the effect of the inciso-cervical position of the retainer was studied. The hypothesis was that retainer material, direction of force, and location of the retainer influenced the force required to cause movement of the tooth.

## 2. Materials and Methods

### 2.1. Tested Materials

The FRC retainers (HS glass (high strength glass), Universal Star Group Limited, Ningbo, China) and two brands of metal retainers—five strands of stainless steel wire (Penta One, 0.55 mm corresponding to 0.0215 inches, Masel, Ortho Organizers, Carlsbad, CA, USA, Lot F1408572) and flattered 8-braided stainless steel wire (Straight 8 Lingual Retainer Wire, 0.71 mm × 0.20 mm corresponding to 0.028 inches × 0.008 inches, Db Orthodontics, Silsden, United Kingdom, Lot no 134378)—were tested using a loading test to determine flexural stiffness of the retainers, which were bonded to the lingual surface of the Frasaco models. There were 7 (n) specimens in each group (Table 1 and Table 2).

**Figure 1:**
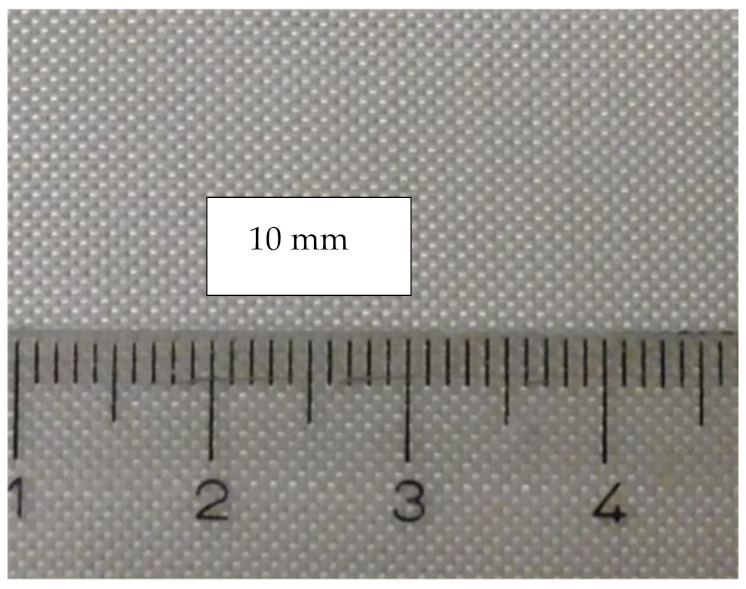
The high-strength (HS glass) fabric with a woven pattern plain was used in the FRC retainer. The direction in the strand for the retainer was 90/0°.

The FRC retainers were made of the bidirectional continuous HS glass fabric, with thickness 0.10 mm, area weight 100 g/m^2^, woven pattern plain, and direction of the fibers 0/90° (Figure 1).

According to the manufacturer, HS glass fiber has a composition of magnesium—alumina—silicate glass similar to that of S-glass, providing 30–40% higher tensile strength than that of E-glass fiber [19].

### 2.2. Production of Retainers

**Figure 2:**
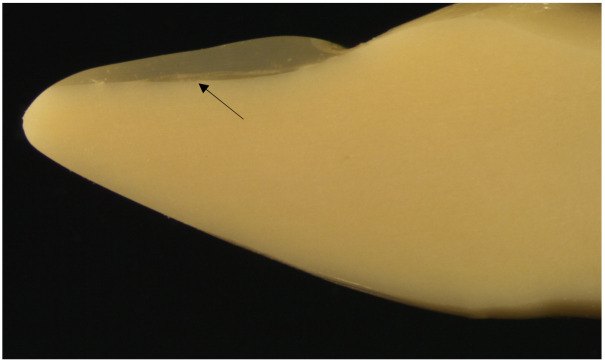
The photograph of the longitudinal section of Frasaco tooth with a bonded FRC retainer and flowable resin composite cover showing the FRC retainer, which is adapted to the surface of the tooth. The arrow points the place of the retainer.

The FRC retainer was impregnated with a resin mixture of 94 weight% bisphenol-A-glycidyldimethacrylate (bis-GMA), 5 weight% of poly(methylmethacrylate) (PMMA), and a 1 weight% photoinitiator system. After impregnation, the FRC retainer was measured with the electronic caliper and cut with scissors into strands of 2 mm wide, approximately 0.07 mm thick, and 24 mm long pieces. The FRC retainer strand and the lingual surface of the plastic canine-to-canine teeth of the Frasaco models (Frasaco GmbH, Tettnang, Germany) were then treated with G-aenial bond, self-etching light-cured adhesive (GC Corporation, Tokyo, Japan, Lot 1108041). The G-aenial bond was first applied to the FRC retainer strand surface and to the teeth surface, left undisturbed for 10 s, and dried for 5 s under air pressure. The piece of polyvinyl siloxane was used to help the placement of the retainer strand on the lingual surface of the plastic teeth from canine to canine (dd.33–43) of the simulation model. The adhesive was light-cured (3 times 20 s) through the siloxane from a 2 mm distance with 3M Espe Elipar S10 (3M, Seefeld, Germany, light intensity 1200 mV, Serial no 939123002122, 210V/50/50 Hz). After removing the siloxane, the FRC retainer was covered by the G-Fix resin composite (GC Corporation, Tokyo, Japan-LOT 1303011) and then light-cured similar to an adhesive without siloxane (Figure 2). Prior retainer placement, one incisor (d.31) of the Frasaco models was shortened from its root analogue by 2 mm to allow for free movement of 0.1 mm in magnitude to occur in the loading test.

**Figure 3:**
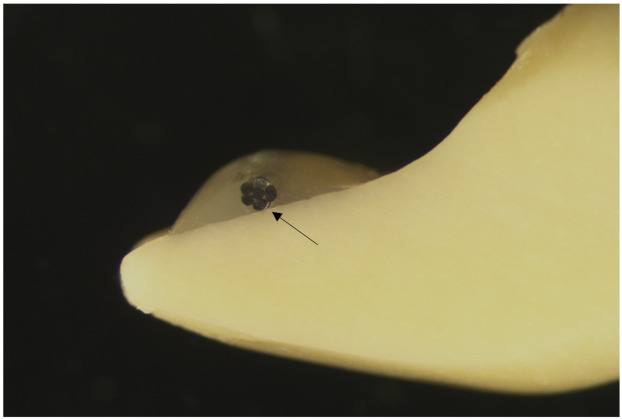
Photograph of the longitudinal section of a Frasaco tooth with Penta One 0.0215 and a flowable resin composite cover. The arrow points the place of the retainer.

**Figure 4:**
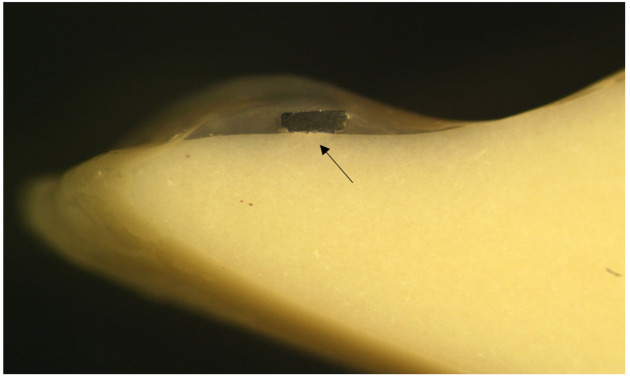
Photograph of the longitudinal section of a Frasaco tooth Straight 8 and flowable resin composite cover. The arrow points the place of the retainer.

The Penta One and the Straight 8 retainers were cut into 24 mm long pieces and formulated so that the retainers fit passively to the lower incisor from canine to canine. Prior to placement of the retainer, the acrylic teeth were treated with G-aenial bond following the manufacturer’s instructions. Next, the metal retainers were set lingually on the lower incisor and canines and the G-Fix resin composite was inserted onto the metal retainer at the center of the tooth so that interdental areas of the teeth were free of the resin composite (Figure 2, Figure 3 and Figure 4).

**Figure 5:**
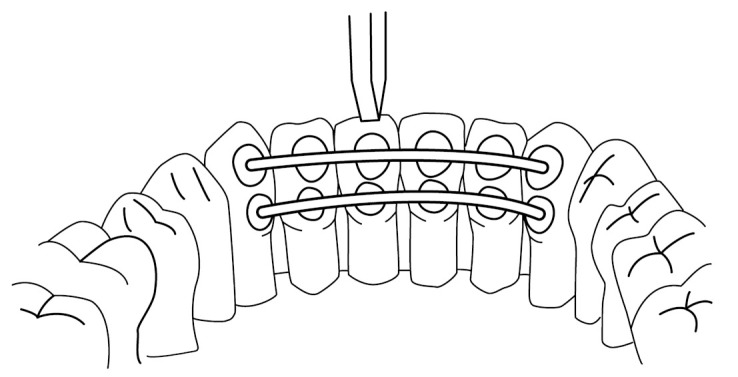
The loading test from the axial direction. In the example, the wires are bonded either 1–2 mm or 3–4 mm from the incisal edge of the teeth.

The resin composite was put on two teeth at a time and then light-cured using the same method as with FRC retainers. All of the retainers were adhered onto two locations of the teeth: 1–2 mm or 4–5 mm from the incisal edges of the teeth (Figure 5). The distance from the margin of the bonding area to the bonding area of the adjacent tooth was ca. 2 mm for the FRC retainer and 4 mm for the metal retainer.

All retainer samples are seen in the photographs. Each photograph was taken from the longitudinal sections of a Frasaco tooth bonded with FRC, Penta One, and Straight 8 retainers and flowable resin composite covers. The photographs were taken using a light microscope (Wild M3Z, Heerbugg Switzerland), Type MDG17, with a magnification of 10 times (Figure 2, Figure 3 and Figure 4).

### 2.3. The Loading Test

**Figure 6:**
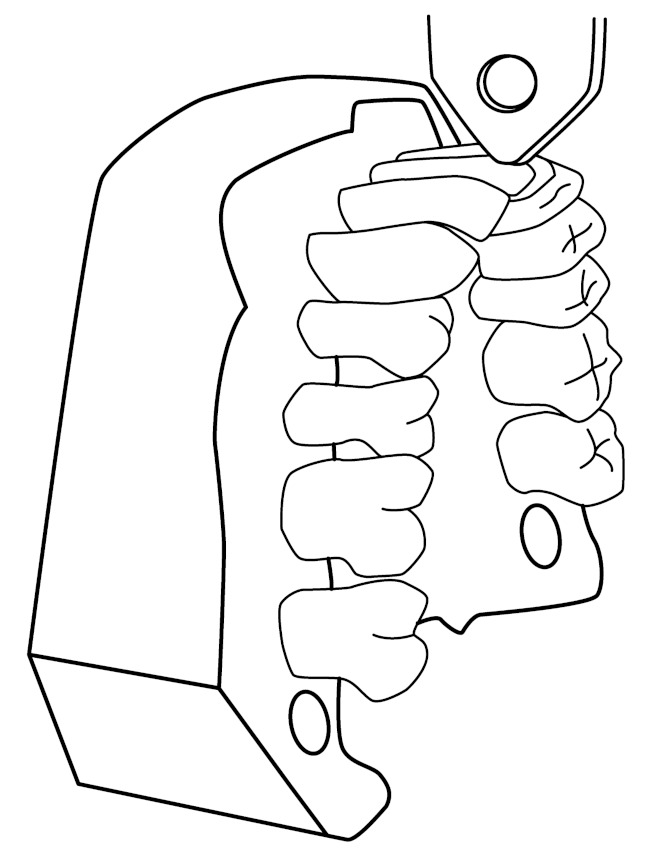
The loading test from the buccal direction.

The unit of measurement for the stiffness of the bonded retainer was the load required to cause movement of 0.1 mm in magnitude of the tooth. The retainers were tested using the loading test in air while dry at room conditions with a universal testing machine with a cross-head speed of 1.0 mm/min (Lloyd LRX30, Lloyd Instruments, Fareman, UK, load cell 2500N) in the simulation model. The loading tip was positioned axially or buccally to obtain a direct force on the incisal edge or labial surface of the teeth d.31 (Figure 5 and Figure 6).

### 2.4. Statistical Analyses

The statistical analysis was carried out using analysis of variance (ANOVA) and SPSS 16.0 (IBM Inc, Armonk, New York, USA) factor analysis (using independent factors: type of retainer, location of the retainer, and direction of force) followed with a post hoc Tukey test (*p* < 0.01). The dependent variable was forced to cause movement of 0.1 mm. Such as in Sfondrini et al. (2017) and Scribante et al. (2019), a deflection of 0.1 mm was selected for this study because clinically vertical teeth movement is very little and the alveolar bone and periodontal ligaments also restrict teeth movement. [20,21].

## 3. Results

**Figure 7:**
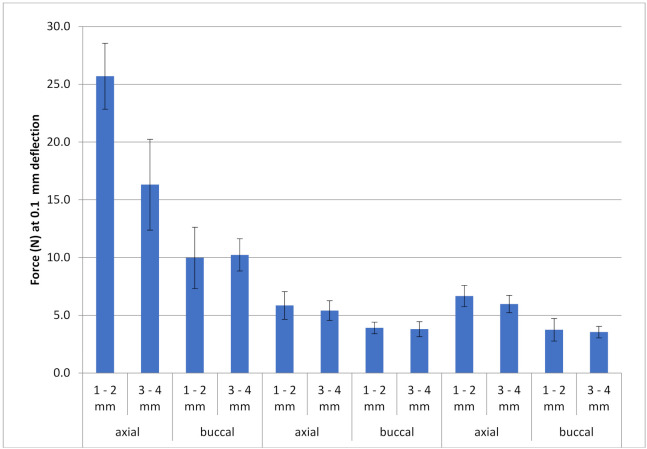
The descriptive results for the load values with standard deviation (vertical bars) at 0.1 mm magnitude of movement of the 12 groups (n = 7) of FRC retainers and metal retainers (Penta One 0.0215 inches and Straight 8 0.028 inches × 0.008 inches retainers). The test was carried out from the buccal or axial directions.

**Figure 8:**
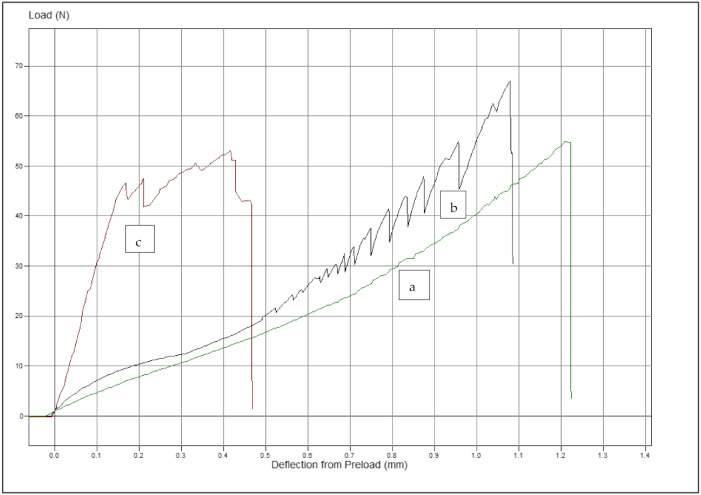
Examples of the load–tooth movement curves: (**a**) metal retainer (Penta One), (**b**) metal retainer (Straight 8), and (**c**) FRC retainer. All retainers were bonded 1–2 mm from the incisal edge of the teeth, and the force was applied from the axial direction. Preload from deflection was 0.05 N.

**Figure 9:**
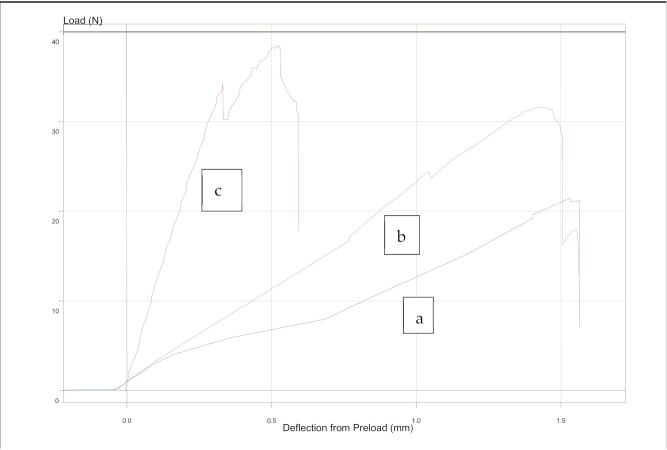
Examples of the load–tooth curves: (**a**) metal retainer (Straight 8), (**b**) metal retainer (Penta one), and (**c**) FRC retainer. All retainers were bonded 1–2 mm from the incisal edge of the teeth, and the force was applied from the buccal direction. Preload from deflection was 0.05 N.

The average load values to move the tooth d.31 in the simulation model of FRC retainer groups were higher than those of metal retainers varying between 10.0 and 25.7 N. The average load values of metal retainer groups varied between 3.6 and 6.7 N. In all retainer types, the load values in the groups were higher when the loading was made from the axial direction than from the buccal direction. When the force was axial, load values were higher in the FRC retainer groups if the retainer was placed closer to the incisal edges (1–2 mm) than in the cervical area (4–5 mm). The differences in the load values are shown in Table 3. The results for the load values of 0.1 mm movement from the measurements of the groups are shown in Figure 7. Examples of the load–tooth movement curves can be seen in Figure 8 and Figure 9.

Analysis of variance (ANOVA) revealed that there was a significant statistical difference between the FRC retainer and the metal retainers (*p* < 0.01) as measured by the loading test. ANOVA also revealed that FRC wire material vs. metal wire had a significant effect (*p* < 0.01) on the position of the wire and the direction of the force. There were no significant differences between metal retainers of Penta One and Straight 8 (Table 3).

## 4. Discussion

Permanent retention is often needed after orthodontic treatment to prevent relapses of the treatment [1,2,4]. However, the lack of space is a problem particularly within the palatinal side of upper incisors [17,22]. In this study, thin FRC retainers were compared with two different steel wires. The material of the retainer, the location of the retainer, and the direction of the force applied were tested on a universal testing machine to demonstrate stiffness of the retainer in a simulated clinical setting. Testing was performed in dry conditions. Wetting of the retainers could possibly have had an effect on stiffness values. It is known that water sorption to the polymer and polymer-based composites reduces material properties [23,24].

Retainers should be flexible enough to allow for physiologic movement of the teeth without failure or debonding [1]. Reynolds assumed that orthodontic attachment should be able to resist 5–8 MPa in strength [25]. The debonding force of the bonded retainers was tested using extracted human teeth, resulting in a debonding force which varied between 5 to 107 N [9,26,27]. In the present study, the retainers were bonded to the plastic incisors of a Frasaco model instead of human teeth. Plastic teeth were selected because same-sized human teeth are difficult to obtain and variation in the enamel structure in natural teeth may have an influence on bond strength [28]. However, Frasaco teeth are made of hard thermosetting plastic and its bonding features are not the same as in natural teeth. In the pilot testing, it was found that, by using a primer, the bond strength of the retainer was high enough for the study design.

Clinically, the stiffness of a retainer is influenced by the material’s modulus of elasticity, thickness, and cross-sectional shape of the device. Based on the literature, the modulus of elasticity of FRC retainers is relatively low. Annousaki et al. (2017) found that FRC retainers have a modulus of elasticity of 5.2–6.9 GPa compared to three-stranded wires at 151–164 GPa [29]. Rucker et al. (2002) showed that multistranded stainless steel wires have a modulus of elasticity of up to 199 GPa [30]. It is important to note that the values presented above are material properties and that, in clinical use, the thickness of the retainer considerably influences the stiffness of the retainer. It was also confirmed in our previous study, which reported that thicker FRC retainers (tex-600 E-glass) had higher maximum load values in bending than thinner (tex-300 E-glass) wires, which were close to the steel wires load values [31]. Alavi et al. (2014) also compared the force levels of 1.2 and 0.75 mm FRC wires and 0.055 mm × 0.071 mm, 0.041 mm × 0.056 mm and 0.7 mm stainless wires in a three-point bending test setup. They noticed that 1.2 mm FRC had significantly higher load values, which corresponded to higher stiffness than other tested wires [32].

In this study, however, it was found that thin (0.07 mm) FRC retainers bonded to the teeth and resisted tooth movement more than the steel retainers, which does not fully support the observation presented in the previous chapter. The results obtained in this study indicate that, besides the material properties, the span length between the bonding spots also have an effect on the retainer’s capacity to withstand occlusal forces. It needs to be noted that the steel retainer was bonded to the teeth by the so-called spot bonding method whereas the FRC retainer was bonded to the entire available bonding surface of the tooth. Therefore, it is obvious that the 2 mm “span” of the FRC material at the region of approximal area required higher force than metal retainer with ca. 4 mm span. Sfondrini et al. showed that the mesiodistal distance between the points of bonding, i.e., the span length of the non-bonded part of the retainer, influenced the mechanics of the retainer–tooth system [20,33]. Thus, based on the results obtained, it can be stated that both the dimensions of the retainer and the mesiodistal span length of the retainer affect the freedom of tooth movement.

The stiffness values between two metal retainers were close to each other in the presently used study design. This was expected and is in line with a study by Sfonfrini et al. who found only minor differences between the metal wires in this respect [20]. Whether the stiffness of the retainer has an impact on the durability of bonding of the retainer to tooth has also been studied. For instance, Bearn et al. noticed that a larger diameter steel wire (0.55 mm; 0.0215 inches) compared to a thinner wire (0.45 mm; 0.0175 inches) provided higher pull out forces to de-bond the wire from the substrate [34]. The result was likely due to the larger bonding surface area rather than being influenced by the wires themselves. Metal wires are modified by a hardening process, and there are so-called dead-soft steel wires, which have lower moduli of elasticity than regular hardened steel. Keeping in mind that the stiffness of the retainer transfers and concentrates stresses between the enamel-bonding resin–retainer system, it can be assumed that this also affects durability of the bonding, as studies by Baysal et al. show [9].

When considering the effect of the direction (buccal vs. axial) of the force used to move the tooth, we found that the magnitude of force from the axial direction was higher than that from the buccal direction in all study groups. An obvious explanation for this is in the cross-sectional geometry of the retainer and its relation to the direction of the force and the stress. When the load is applied from the buccal direction, the cross-sectional dimension (thickness) is considerably less than when the direction is axial and the height of the retainer in the approximal tooth space resists the stress.

The magnitude of force was lower in FRC retainers, which were bonded at 3–4 mm from the incisal edge of the tooth rather than from 1–2 mm. However, more lingually bonded FRC retainers may neither be more durable nor resist debonding better. In any case, force levels in these retainers were considerably higher than in steel retainers. The disadvantage of more lingually bonded retainers is also that teeth cleaning may be more challenging, which may increase plaque and calculus accumulation.

## 5. Conclusions

It was found that there was a significant difference between FRC and metal retainers´ stiffness in the simulated clinical setting. The results of this study suggest that spot-bonded metal retainers had lower stiffness and allowed for tooth movement with a lower magnitude of force than FRC retainers, which were bonded from the entire lingual surface of the tooth. This likely has an impact on the durability of the bond of the retainer. FRC retainers are an alternative option to metal retainers for metal-allergic patients and in aesthetic cases. However, metal retainers are more commonly used since placement of multistranded steel retainers is a relatively simple process for orthodontists, yet the placement of FRC retainers may be more difficult. FRC retainers need to be investigated and developed more to achieve better and more useful FRC retainers.

## Figures and Tables

**Table 1:** Test groups according to the retainer material, direction of the applied load, and position of the retainer, with seven samples in each group.

Group	Position of the Retainer	Force Direction	Retainer
1.	1–2 mm from the incisal edge	axial	FRC retainer
2.	4–5 mm from the incisal edge	axial	FRC retainer
3.	1–2 mm from the incisal edge	buccal	FRC retainer
4.	4–5 mm from the incisal edge	buccal	FRC retainer
5.	1–2 mm from the incisal edge	axial	Penta One
6.	4–5 mm from the incisal edge	axial	Penta One
7.	1–2 mm from the incisal edge	buccal	Penta One
8.	4–5 mm from the incisal edge	buccal	Penta One
9.	1–2 mm from the incisal edge	axial	Straight 8
10.	4–5 mm from the incisal edge	axial	Straight 8
11.	1–2 mm from the incisal edge	buccal	Straight 8
12.	4–5 mm from the incisal edge	buccal	Straight 8

**Table 2:** Tested materials and their composition.

	Round Metal Retainer	Flat Metal Retainer	Fiber Reinforced Composite
Name	Penta One 0215	Straight 8	FRC net wire
Manufacturer	Masel, Ortho Organizers	Db Orthodontic	Universal Star Group
Design	Coaxial 5 wires	Braided 8 wires	woven pattern plain
Material	Stainless steel	Stainless steel	HS glass fabric
Dimensions (mm)	0.55 mm × 24 mm	0.71 mm × 0.20 mm × 24 mm	0.07 mm × 0.07 mm
Bonding technique	in one spot	in one spot	wholly covered by adhesive
Impregnation	no	no	bis-GMA 94%, PMMA 5%, photoinitiator system 1%

bis-GMA = bisphenol-A-glycidyldimethacrylate, PMMA = poly(methylmethacrylate).

**Table 3:** Load values (in N) required to cause 0.1 mm movement of tooth d.31 with retainers of different materials, positioned from two distances from the incisal edge (1–2 mm; 3–4 mm) and loaded axially and buccally. A different superscript letter (small letter within row, capital letter within column) above a value indicates statistically significant differences (*p* < 0.05, Tukey).

Force Direction				
	Axial		Buccal	
Retainer Material				
	1–2 mm	3–4 mm	1–2 mm	3–4 mm
FRC retainer	25.7 (2.9) ^aA^	16.3 (3.9) ^bA^	9.975 (2.7) ^cA^	10.2 (1.4) ^cA^
Penta one	5.9 (1.2) ^aB^	5.4 (0.9) ^aB^	3.9 (0.5) ^bB^	3.8 (0.7) ^bB^
Straight 8	6.7 (0.9) ^aB^	6.0 (0.8) ^aB^	3.7 (1.0) ^bB^	3.6 (0.5) ^bB^

## Data Availability

Data available upon request from the corresponding author.
